# Quantum face recognition protocol with ghost imaging

**DOI:** 10.1038/s41598-022-25280-5

**Published:** 2023-02-10

**Authors:** Vahid Salari, Dilip Paneru, Erhan Saglamyurek, Milad Ghadimi, Moloud Abdar, Mohammadreza Rezaee, Mehdi Aslani, Shabir Barzanjeh, Ebrahim Karimi

**Affiliations:** 1 Department of Physics and Astronomy, Institute for Quantum Science and Technology, University of Calgary, Calgary, AB T2N 1N4 Canada; 2 BCAM - Basque Center for Applied Mathematics, Alameda de Mazarredo 14, 48009 Bilbao, Basque Country Spain; 3 Nexus for Quantum Technologies, University of Ottawa, 25 Templeton Street, Ottawa, ON K1N 6N5 Canada; 4 Department of Physics, University of Alberta, Edmonton, AB T6G 2E1 Canada; 5 Department of Physics, Isfahan University of Technology, Isfahan, 8415683111 Iran; 6 Institute for Intelligent Systems Research and Innovation (IISRI), Deakin University, Geelong, Australia; 7 National Research Council of Canada, 100 Sussex Drive, Ottawa, ON K1A 0R6 Canada

**Keywords:** Applied optics, Quantum physics

## Abstract

Face recognition is one of the most ubiquitous examples of pattern recognition in machine learning, with numerous applications in security, access control, and law enforcement, among many others. Pattern recognition with classical algorithms requires significant computational resources, especially when dealing with high-resolution images in an extensive database. Quantum algorithms have been shown to improve the efficiency and speed of many computational tasks, and as such, they could also potentially improve the complexity of the face recognition process. Here, we propose a quantum machine learning algorithm for pattern recognition based on quantum principal component analysis, and quantum independent component analysis. A novel quantum algorithm for finding dissimilarity in the faces based on the computation of trace and determinant of a matrix (image) is also proposed. The overall complexity of our pattern recognition algorithm is $O(N\,\log N)$—*N* is the image dimension. As an input to these pattern recognition algorithms, we consider experimental images obtained from quantum imaging techniques with correlated photons, e.g. “interaction-free” imaging or “ghost” imaging. Interfacing these imaging techniques with our quantum pattern recognition processor provides input images that possess a better signal-to-noise ratio, lower exposures, and higher resolution, thus speeding up the machine learning process further. Our fully quantum pattern recognition system with quantum algorithm and quantum inputs promises a much-improved image acquisition and identification system with potential applications extending beyond face recognition, e.g., in medical imaging for diagnosing sensitive tissues or biology for protein identification.

## Introduction

In any intelligent image processing system, there are essentially two main steps: the acquisition of the image and the recognition of the desired patterns. Image acquisition for any pattern recognition method can be performed in multiple ways. For instance, classical sources (incoherent light from thermal radiation or a coherent beam from a laser) or quantum sources (entangled photons obtained from down conversion or squeezed light) can be used to obtain the images. Classical bright field imaging techniques employing the former sources, have the disadvantage of high probe illumination requirement, especially while imaging sensitive samples. Additionally, they are also plagued by the shot noise inherent in the intensities, and the background noise from the environment. Quantum techniques such as quantum illumination, or ghost imaging or even interaction-free imaging, alleviates the problems of background noise, and the probe illumination by utilizing quantum correlations between photon pairs^1,2^. Furthermore, quantum sub-shot noise imaging^3^ and super resolution techniques^4^ enhance the noise sensitivity and resolution in any images beyond the classical limits.

**Figure 1:**
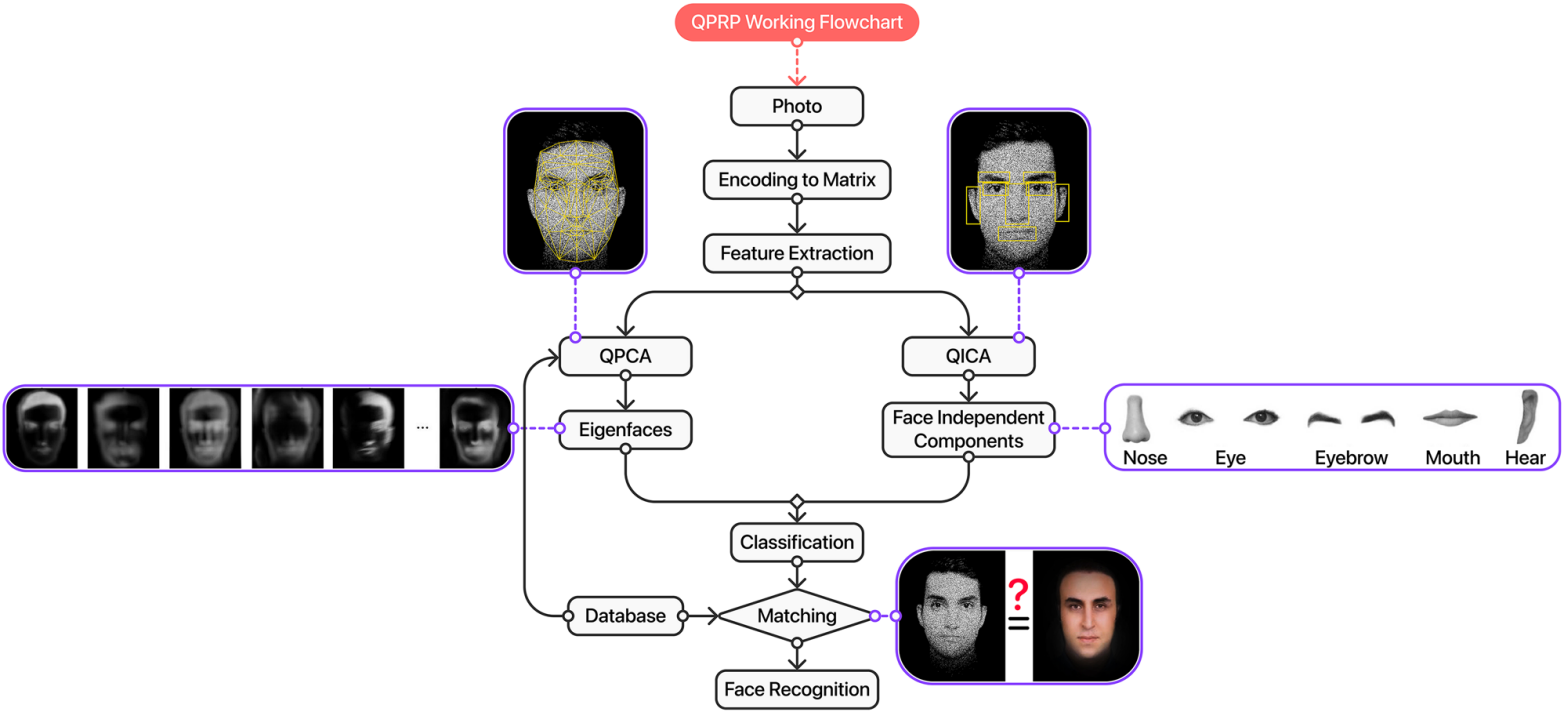
Flowchart of the quantum algorithm for face recognition. The quantum algorithm is proposed to be performed in a quantum processor, which we call it quantum pattern recognition processor (QPRP). First the image is converted into matrix form, on which feature extraction algorithms such as quantum principal component analysis (QPCA) or quantum independent component analysis (QICA) are applied. QPCA extracts the eigenstates (or eigenfaces) of the covariance matrix of the images in the database. The eigenfaces include information like average face, gender (male, female), face direction, brightness, shadows, etc. QICA extracts the independent elements such as eyes, eyebrows, mouth, nose, etc. in a face. The complexity of this stage is O($\log {N})$—*N* is the dimension of th image. Then, the given faces are compared with the faces in the database by using dissimilarity measure based on the log determinant divergence, and the best match among the faces in the database is identified.

As a second important step, pattern recognition in the acquired images is a prominent feature of any intelligent imaging system. Face recognition^5,6^ is one of the branches of pattern recognition, with numerous applications such as face ID verification, passport checks, entrance control, computer access control, criminal investigations, crowd surveillance, and witness face reconstruction^7^, among several others. For face recognition, several classical machine learning algorithms exist^8^, generally requiring huge computational resources especially when faced with the problem of identification from a large database. Quantum machine learning algorithms employing quantum features such as superposition and entanglement^9,9–17^ promise enhancements in terms of the computing resources and the speed compared to the classical counterparts. Several experimental researches have been done to implement these algorithms^18–25^. In this article, we present a quantum algorithm for face recognition as one of the potential applications of quantum algorithms in machine learning.

The problem of identification of faces from any images generally constitutes different steps (shown in Fig. 1): creating a database of faces consisting of training and test images, feature extraction using principal component analysis (PCA), linear discriminant analysis (LDA) or independent component analysis (ICA), feature matching using dissimilarity measures, and recognition^26^. PCA extracts the eigenstates (or eigenfaces) of the covariance matrix of the images in the database, including information like average face, gender (male or female), face direction, brightness, shadows, etc. ICA, however, extracts the independent elements such as eyes, eyebrows, mouth, nose, etc. in a face. Quantum algorithms which provide speedup for PCA and ICA have already been proposed^9,27^. Here, we focus on three main steps: (1) Quantum Principle Component Analysis (QPCA)^9^, (2) Quantum Independent Component Analysis (QICA)^27^, and (3) Dissimilarity measures (i.e., face matching), to develop a quantum algorithm for face recognition. In what follows, we present a quantum algorithm for dissimilarity measures for face matching with speedup. This is based on a quantum algorithm to compute the log determinant divergence using both the determinant and the trace of a matrix. Our algorithm combined with the inputs obtained from quantum imaging techniques provides a fully intelligent pattern identification system, with the joint benefit of the low-dose and higher resolution of quantum imaging methods, and the speedup and efficiency of the quantum algorithms. Figure 1 shows the flowchart of the quantum algorithm for the pattern identification.

**Figure 2:**
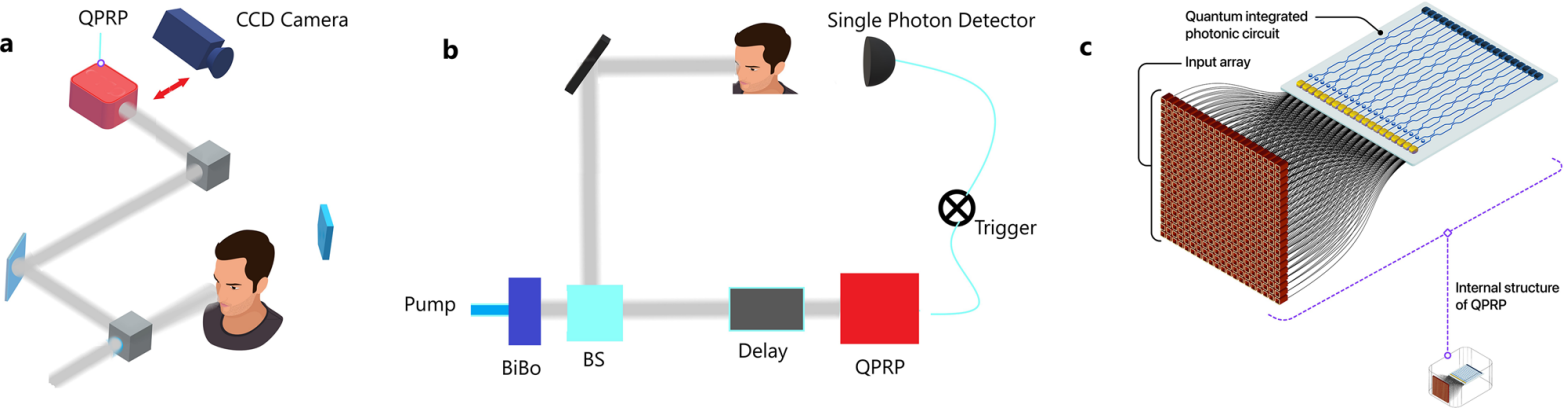
Intelligent pattern recognition in quantum imaging. Data from quantum imaging methods such as (**a**) interaction free imaging and (**b**) ghost imaging act as an input to (**c**) quantum pattern recognition processor (QPRP). The latter, i.e., QPRP, applies quantum machine learning to find the patterns in the database.

## Quantum face recognition

Classical algorithms are unable to process quantum data directly. During the conversion of the quantum states (qubits) to classical data (bits), most of the information is lost in the measurement process, due to the “collapse” of the wavefunction. Although techniques such as quantum state tomography implemented on unlimited ensemble of the states can be used to fully reconstruct the quantum states from classical projections, these processes are generally complex and expensive. Therefore, the optimal input to our quantum algorithms, would be the quantum states directly obtained from quantum processes, for example, quantum imaging methods, or from a quantum memory, without performing a strong measurement on the wavefunction.

Photonic quantum memories^28^, allowing storage and on-demand retrieval of quantum states of light, is one of the key components for the realization of quantum optical pattern-recognition technology. Quantum memories essentially form a quantum database for the matching stage in the recognition process. With the state-of-art quantum memories, the possibility of storing hundreds of spatial modes has already been shown in experimental studies using atomic-cold gases^29,30^. Furthermore, using solid-state atomic memories, it is possible to simultaneously store hundreds of photonic quantum states in distinct temporal modes, thus allowing us to store patterns scanned at separate times^31,32^. In addition, optically accessible spin-states of certain atomic systems can reach several hours of coherence time^33^. A very recent experimental demonstration reports one-hour memory lifetime for light storage, showing the feasibility of long-lived photonic quantum memory devices^34^. Atomic memory approaches have also been shown to reach high retrieval efficiencies up to $92\%$^35^ and high fidelities above $99\%$^36^. However, an implementation with all of the aforementioned properties still remains as a challenge in developing a practical quantum database memory.

**Figure 3:**
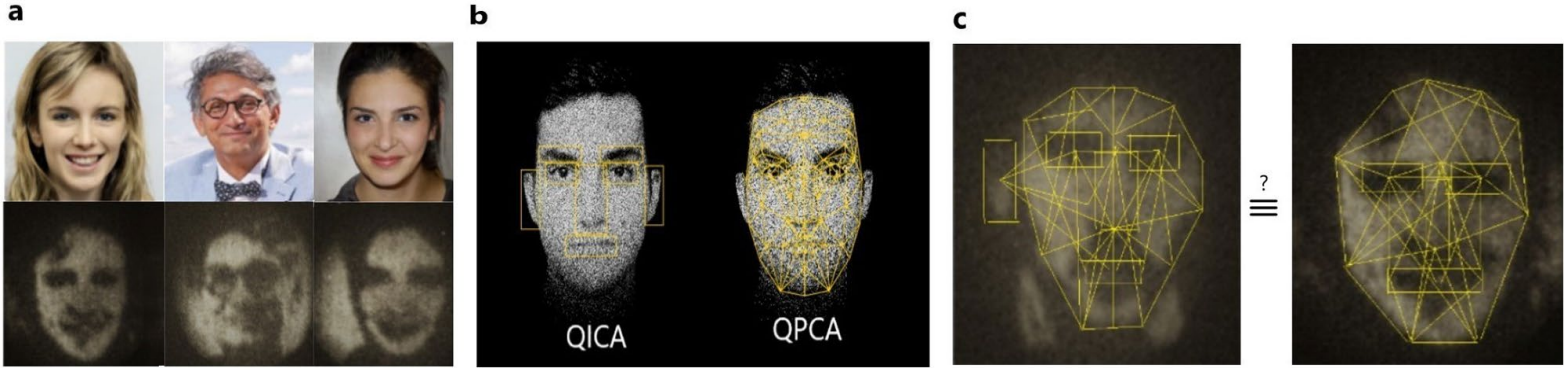
Face recognition in ghost images. (**a**) Images of the original human faces (top) and the corresponding experimental ghost images (bottom) obtained in a ghost imaging setup. A femtosecond laser is used to generate spatially entangled photon pairs. One of the photons illuminates a spatial light modulator, which imprints different images onto the photon, and can act as a trigger for the other photon that was detected by an intensified CCD camera. Each of the images was obtained by the accumulation of 300 frames with an exposure time of 0.5s, which translates to a run time of 150s. (**b**) Quantum Independent Component Analysis (QICA), and Quantum Principal Component Analysis (QPCA), of the faces to detect the independent components, and principal features in the faces. (**c**) Dissimilarity measure between the ghost images with the images in the database for their identification. As per the copyright policies of the journal, in this illustration we use artificially generated faces (the first and the third) from the website https://www.thispersondoesnotexist.com. For the second figure, the coauthor provided the required consent for both the experiment and use in the manuscript.

Quantum techniques such as quantum ghost imaging^37^, quantum lithography^38^, or quantum sensing^39^, when appropriately interfaced with photonic quantum processors, for example an array of optical fibers connected to an integrated quantum photonic circuit, can also act as inputs to our algorithms (see Fig. 2). Here for the case of our face recognition algorithm, we assume that the input images are acquired by quantum ghost imaging^37^. Ghost imaging exploits the spatial correlations between photon pairs generated through a nonlinear process called spontaneous parametric down-conversion (SPDC). Since the images are obtained by triggering the shutter in order to capture only the “coincident” photon pairs, the level of background noise is significantly reduced, along with a reduction in probe illumination. In a variation of this technique using non degenerate photon pairs, the image detection and sample interaction can happen at different wavelengths, which can be useful when imaging sensitive tissues when limited in detection technologies^40^. Combining quantum detection techniques such as interaction-free measurement with ghost imaging, the illumination level required for the same levels of Signal to Noise ratio (SNR) in images^41^ is further reduced significantly. Figure 3 shows some of the images of human faces obtained in a quantum ghost imaging setup, where spatially correlated photon pairs (namely signal and idler), are generated by pumping a BiBO crystal with pump photons. Phase holograms placed in a Spatial Light Modulator, a liquid crystal device, created by superimposing the human faces with a diffraction grating acts as an object for the signal photon, while the idler photon passes to the Intensified Charged Coupled Devices (ICCD) camera via a delaSupplementary Informationy line. The images are obtained by triggering the ICCD shutter with the signal photons detected through a Single Photon Avalanche Diode (SPAD) detector—see (SI) for the detail of the experimental setup.

### Quantum principal component analysis (QPCA)

We have now the input images either retrieved from a quantum memory or directly as outputs from a quantum imaging setup. The pattern recognition processor applies Quantum Principal Component Analysis (QPCA)^9,42^ to extract the principal eigenvectors of the covariance matrix $C_X$, formed by the set of the training images.

Let us consider a set of *N*-dimensional training images (or faces), $\{\vert {x}^{(1)} \rangle ,\ldots , \vert {x}^{(M)} \rangle \}$. Here, $\vert {x}^{(i)} \rangle$ is the i-th training image, which is given by,

1 $$\begin{aligned} \vert {x}^{(i)} \rangle =\sum _{q =1}^{N} x_q^{(i)} \vert {\psi }^{(i)}_q \rangle , \end{aligned}$$

where $x_q^{(i)}$ are the components, and $\vert {\psi }^{(i)}_q \rangle$ are the basis kets. The covariance matrix ${C}_X$ can be formed as a sum over *M* training faces^42^,

2 $$\begin{aligned} {C}_X = \frac{1}{M} \sum _{i=1}^{M} \vert { x}^{(i)} \rangle \langle { x}^{(i)} \vert . \end{aligned}$$

The next step is to exponentiate the covariance matrix ${C}_X$, so that we can use the Quantum Phase Estimation (QPE) subroutine for finding the eigenvectors and eigenvalues. It has been shown that the exponentiation of the covariance matrix, i.e., $e^{-i{C}_X\,t}$, can be performed in ${\mathcal {O}}(\log {}N)$ time^9^.

In QPCA algorithm, for the phase estimation subroutine, we apply the operator ${U} = e^{-i{C}_X\, t}$ on ${C}_X$^42^. The action of *U* on one of the states $\vert x^{(i)}\rangle$ in ${ C}_X$ is:

3 $$\begin{aligned} e^{-i{C}_X\,t} \vert x^{(i)} \rangle \rightarrow \sum _{j=1}^{M} c^{(ij)} \vert {\phi }^j \rangle , \end{aligned}$$

where $\vert {\phi }^{j} \rangle$’s are the eigenvectors of ${C}_X$, and $c^{ij}=e^{-i{\tilde{\lambda }}_c^{(j)}t}\langle \phi ^j \vert {x}^{(i)} \rangle$ in which ${\tilde{\lambda }}_c^{(j)}={\left( 2\pi \lambda _c^{(j)}t\right) }/{2^n}$ where $\lambda _c^{(j)}$’s are the corresponding estimated eigenvalues of ${C}_X$ with precision *n*^9,42^.

In order to obtain the principal eigenfaces (the eigenvectors of the covariance matrix with larger eigenvalues), we define a score $s^{(ij)}$, which is the projection of an eigenvector $\vert \phi ^{(j)} \rangle$ on a training vector $\vert x^{(i)} \rangle$,

4 $$\begin{aligned} s^{(ij)}=\langle x^i \vert \phi ^j \rangle =\sum _{q=1}^{N}x^{(i)}_q \phi ^{(j)}_q, \end{aligned}$$

where $\phi ^{(j)}_n$ are the components of the eigenvector $\vert \phi ^j \rangle$. The eigenvectors corresponding to the *r* highest scores are the principal components (or eigenfaces). Each face can be expanded in terms of the *r* eigenfaces (principal components) but with different weights $\omega _j'$s as follows

5 $$\begin{aligned} \vert \text {Face}^{(i)} \rangle = \sum _{j=1}^{r} \omega _j \vert {{\varvec{\phi }}}^j \rangle . \end{aligned}$$

The “mean image” is the eigenface corresponding to the largest eigenvalue of $C_X$. The QPCA algorithm is efficient for the case $r\ll N$^42^.

### Quantum independent component analysis (QICA)

In classical machine learning, Independent Component Analysis (ICA) is performed to decompose an observed signal into a linear combination of unknown independent signals^26^. Similar to the PCA, the ICA finds a new basis to represent the data, however with a different goal. We assume that there is a data set of faces $s \in {R}^d$ that is a collection of *d* independent elements in the face such as nose, eye, eyebrow, mouth, etc. Each image observed through a camera can be expressed as ${x}={F}\cdot s$, where *F* is a mixing matrix of the independent face elements. Repeated observation gives us a dataset *x* as $\{x^{(i)},\ldots ,x^{(M)}\}$, and ICA estimates the independent sources $s^{(i)}$ that had generated the face. We let ${W}={F}^{-1}$ which is the unmixing matrix and solve the linear systems of equations $s^{(i)}={W}\,x^{(i)}$ for estimating the independent elements of the face. We should note here that $s^{(i)}$ is a d-dimensional vector and $s^{(i)}_j$ is the data of element *j*. Similarly, $x^{(i)}$ is an d-dimensional vector, and $x^{(i)}_j$ is the observed (or recorded) element *j* by camera. The ICA can be exponentially speedup via a quantum algorithm for sparse matrices, with the Harrow-Hassidim-Lloyd (HHL) algorithm^27^, which is used to solve linear systems of equations optimally with $O(\log {}N)$. For comparison, classically it takes a time $O(N^3)$ to be solved via the Gauss elimination, and approximately $O(N\sqrt{\kappa })$ via iterative methods^27^ for a sparse matrix of size $N\times N$, with $\kappa$ being the ratio between the greatest and the smallest eigenvalue.

### Pattern matching: comparing faces

As important details of a face are obtained either by using QPCA or QICA, each face is represented in the form of a sparse matrix in which non-important elements are set to zero. The last and important step of the algorithm is comparing the face patterns to recognize the target face. Pattern matching algorithms investigate exact matches in the input with pre-existing patterns in the database. In fact, the problem here is comparing matrices with each other. The evaluation of matching between matrices (or face patterns) can be done by using “dissimilarity”^43^ measures that calculate the “distance” between the matrices. The lower the values of the dissimilarity/distance measures, more similar the matrices, with the fully matched matrices having a zero distance. One such distance measure used to compare two matrices *X* and *Y* is called the “Log-determinant divergence”^43,44^ defined as,

6 $$\begin{aligned} D(X,Y)=\text {Tr}\left( X\cdot Y^{-1}\right) -\log \det \left( X\cdot Y^{-1}\right) -N, \end{aligned}$$

where *N* is the dimension of the matrices. When $D=0$, the matrices *X* and *Y* are completely matched, and higher the distance value the more different are the matrices. The least value among the all distance values identifies the best match and consequently recognizes the face. As it is seen in the distance formula, it is a benefit to be able to calculate the trace and the determinants of matrices with speedup to expedite the distance calculation. In the following, we propose quantum algorithms for computation of the determinant and the trace of a sparse matrix.

#### Quantum computation of sparse matrix determinants and trace

To obtain a measure of dissimilarity between two matrices we need to calculate the determinant and the trace of the sparse matrix $A = X\cdot Y^{-1}$. First we calculate $Y^{-1}$ using the HHL algorithm^27^ and obtain *A* by multiplying it with *X*. We then apply the Quantum Phase Estimation (QPE) subroutine, which consists of a quantum Fourier transform (QFT) followed by a controlled Unitary ($\text {CU}$) operation, with $U = e^{-iA\,t}$, and a inverse quantum Fourier transform. We then apply a controlled Rotation operation followed by the inverse Quantum Phase Estimation (QPE) subroutine. At the end we have a multiplication operator $\Pi$ which finally gives us the product of the eigenvalues—the algorithm steps are explained in more detail in the Supplementary Information. The running time of the algorithm up to the third step, i.e. applying the controlled-U operator, is $O(\log {}N(s^2 \kappa ^2 /\epsilon ))$^27^, where *s* is the sparsity, $\kappa$ is the ratio of largest eigenvalue to the smallest eigenvalue of *A*, and $\epsilon$ is the acceptable error. Additionally, the multiplication operation in the last step can be performed in time $O(\log {} N)$ and the algorithm should run *N* times. Therefore, the overall complexity of the algorithm is $O\left( N\,{\log {}}N(1+ s^2 \kappa ^2 / \epsilon )\right)$, which is much faster than the classical ones (see Table 1).

**Table 1:** A Comparison of complexities between the classical approaches and our quantum approach, current work (CW), for the computation of determinant.

Approach	Method	Complexity	References
Classic	Laplace	$N^3$	^45^
Classic	Gaussian	$N^3$	^45^
Classic	Coppersmith–Winogard	$N^{2.373}$	^46^
Classic	Wiedemann	$N^{2} \log N$	^47^
Quantum	Our method	$N \log N$	CW

In order to compute the trace of the matrix *A*, an adder quantum algorithm^48^ can speedup the computation. The adder operation between two diagonal elements is mainly based on the quantum Fourier transform (QFT), i.e. $\vert \Phi (a) \rangle :=\text {QFT}\,\vert a \rangle = \frac{1}{\sqrt{N}}\sum _{k=0}^{N-1}e^{i\frac{2\pi a k}{N}}\vert k \rangle$ and the inverse QFT, i.e., $\text {QFT}^{-1}\vert \Phi (a) \rangle =\vert a \rangle$. By continuation of this method sequentially for the all diagonal elements, one can obtain the trace of the matrix. The detail of the adder algorithm and the quantum circuit for the computation of trace is discussed in the Supplementary Information (Fig. S2 shows the corresponding quantum circuit). The whole process which is based on QFT and $\hbox {QFT}^{-1}$ has a complexity of $O(\log {} N)$ (Table 2).

**Table 2:** Summary of estimated complexities in quantum face recognition algorithm.

Method	Output	Complexity	References
QPCA	Eigenfaces	$\log N$	^9^
QICA & HHL	Face components	$\log N$	^27^
HHL	Matrix inversion	$\log N$	^27^
Our method	Determinant calculation	$N \log N$	CW
Our method	Trace calculation	$\log N$	CW
Log-det divergence	Face matching	$N \log N$	CW
Our method (General)	Face recognition	$N \log N$	CW

QPCA and QICA both have logarithmic complexities, i.e., $O(\log {}N)$. For the calculation of the log determinant divergence, the computation of trace has a complexity of $O(\log {}N)$, while the determinant has complexity of $O(N\log {}N)$). Hence, the overall complexity of the whole algorithm is $O(N\log {} N)$. Table 2 shows a summary of estimated complexities along with the complexity of the general quantum face recognition algorithm.

## Discussion

Here, we have shown that classically the best pattern recognition algorithm based on ICA and PCA would take at least O($N^2$Log*N*) steps while our algorithm only takes O(*N*Log*N*) steps, which is an order of magnitude faster—*N* is the dimension of images (see Table 3 for some estimates). We should note here that our protocol is not compared with neural networks approaches, for instance, we did not compare our quantum protocol with a classical deep learning model. In fact, the protocol suggested in our paper is only a comparison with its classical counterparts based on classical PCA, ICA, and Distance. There are other quantum models which are robust versus their classical counterparts, for example, using QPCA and quantum neural networks show advantages over the best classical approaches^49,50^, or training deep quantum neural networks give a fast optimization and a striking robustness to noisy training data^51^. It has been shown that a quantum convolutional neural network uses only O(Log*N*) variational parameters for input sizes of *N* qubits, allowing for its efficient training and implementation on realistic, near-term quantum devices^52^.

Apart from these, our protocol would also benefit from the quantum nature of the image acquisition, namely lower sample exposure, background acquisition, and resolution enhancement. From the acquisition point of view, to have an image with a desirable resolution the images were accumulated for 300 frames with an exposure time of 0.5 s, hence, to obtain a single image it takes roughly 150 s, i.e. independent from the image processing side (see Table 4). Classical or direct imaging offers much faster image acquisition times, however here we are relying on the entangled photon pairs, the production rate of which is less than 1% of the total power of the pump beam, hence we have much less photons interacting with the sample (or face) and hence the need for longer exposure times. In fact, it is not the speed that is the improvement in the imaging part. With higher pump power and efficient crystals this process can also be improved further however the state of the art on this would not match the acquisition speed of classical methods. The advantage of this is that we achieve a better SNR if we compare the images with classical acquisition with a similar number of photons interacting with the sample.

Depending on the database, compared to the best classical pattern recognition algorithm, the proposed quantum algorithm will be *N* times faster. Both the QPCA and QICA can be used for face recognition in classical images as well, as it can perform the pattern identification on any matrix. Run on a quantum processor, it would provide a speedup in the process compared to the traditional recognition methods. At the moment, due to the image size, no quantum computers are yet available that can successfully implement the quantum protocol. However, this is indeed a possible future step once such devices are available.

**Table 3:** Numerical estimates of the time complexities (in arbitrary units) for the classical and the quantum protocol for different input image dimensions along with the corresponding number of qubits required for encoding.

Image dimensions	Encoding qubits	Classical processing time (in a.u.)	Quantum processingtime (in a.u.)
$64\times 64$	6	7398.1	115.5
$128\times 128$	7	34524.5	269.7
$256\times 256$	8	157826.4	616.5
$512\times 512$	9	710218.8	1387.1
$1024\times 1024$	10	3156528.2	3082.5
$2028\times 2028$	11	13888724.4	6781.6

**Table 4:** Numerical estimates of the Signal to Noise ratio (SNR) in the ghost images of different dimensions along with the total number of photons acquired corresponding to different acquisition times.

Image dimensions	Total photons = Average photons/pixel $\times$ no of pixels	Imaging time (sec)	Signal to noise ratio (SNR)
64$\times$64	$98304 = 12 \times (64)^2$	4	3.46
64$\times$64	$786432 = 96 \times (64)^2$	32	9.80
64$\times$64	$3686400 = 450 \times (64)^2$	150	21.21
128$\times$128	$14745600 = 450 \times (128)^2$	150	21.21
256$\times$256	$58982400 = 450 \times (256)^2$	150	21.21
512$\times$512	$6291456 = 450 \times (512)^2$	150	21.21
1024$\times$1024	$235929600 = 450 \times (1024)^2$	150	21.21
2028$\times$2028	$3774873600 = 450 \times (2028)^2$	150	21.21

## Conclusion

In summary, we propose a new concept of a quantum protocol for 2D face recognition, combining the benefits of quantum imaging in image acquisition with the speedup from the quantum machine learning algorithms. In this concept, we consider images to be obtained via a ghost imaging protocol either as inputs to the quantum memories or as a hardware encoding of quantum information for the photonic pattern recognition processor. Feeding the “images” directly from a quantum protocol also eliminates the need for the conversion of classical data to quantum inputs for the processor saving valuable computational resources. The quantum pattern recognition processor then runs an algorithm composed of three main subroutines: (1) quantum principal components analysis (QPCA), (2) quantum independent component analysis (QICA), and (3) quantum dissimilarity measures for comparing faces. For the QPCA and QICA, we propose slight modifications in the existing algorithms, whereas for finding the dissimilarity measure, we propose a novel algorithm for obtaining the distance between two matrices based upon a metric called log-determinant divergence. Our algorithm obtains the determinant and the trace of the two matrices in $O(N\log {N})$ time—*N* is the dimension of the matrix. Complexity analysis shows that all of the three parts have speedup as compared to their classical counterparts, with the overall complexity given by $O(N\log {N})$. Our conceptual protocol provides a framework for an intelligent and fully quantum image recognition system with quantum inputs and a quantum machine learning processor. The joint benefits of the quantum image acquisition and quantum machine learning promises exciting technological developments in the field of image recognition systems.

## Supplementary Information

Supplementary Information.

## Data Availability

All data needed to evaluate the conclusions in the paper are present in the paper and/or the Supplementary Materials. Additional data related to this paper may be requested from V.S.(vahid.salari1@ucalgary.ca) or E.K. (ekarimi@uottawa.ca).
