# Supplementary material for: Quantum face recognition protocol with ghost imaging
Source: Sci Rep. 2023 Feb 10;13:2401. doi: 10.1038/s41598-022-25280-5 (PMC9918728; doi:10.1038/s41598-022-25280-5)
Supplement: Supplementary file 1 — Supplementary Information. [file 41598_2022_25280_MOESM1_ESM.pdf]

Supplementary Information for: Quantum Face Recognition Protocol with Ghost Imaging

S1. QUANTUM IMAGING

We elaborate on the experimental details of the image acquisition for the quantum pattern recognition protocol. Spatially correlated photon pairs, usually called signal and idler photons, are generated from a Spontaneous Parametric Down Conversion Process (SPDC) by pumping a nonlinear crystal. Utilizing the position and momentum correlations in these down converted photon pairs, one can non-locally obtain an image of an object that interacted only with the idler photons. The experimental setup we use is similar to a conventional ghost-imaging setup, see Fig. S1, with our object being a hologram placed in a Spatial Light Modulator (SLM), a liquid crystal device. We use a 1 GHZ, 100 fs pulsed laser to pump a nonlinear crystal, β-Barium Borate (BBO), for generating a second harmonic output. We then use the second harmonic beam to pump a Type-I bismuth triborate (BiBO) crystal for the down conversion of photon pairs. The generated signal and idler pairs are split into two paths, i.e. the object arm (idler) and the camera arm (signal), via a 50:50 Beamsplitter (BS). The idler photon interacts with the SLM, on which we display the holograms created by superimposing the original face image with a diffraction grating. The grating sends only the desired photons from the incident beam to the the first order, which then are coupled to a Single Mode Fibre (SMF) and sent into a Single Photon Avalanche Diode (SPAD) detector which can be used to trigger the collection of the photons in the Intensified CCD (ICCD) camera. The images obtained that are shown in Fig. 2 were taken with 0.5 s exposure accumulated over 300 frames.

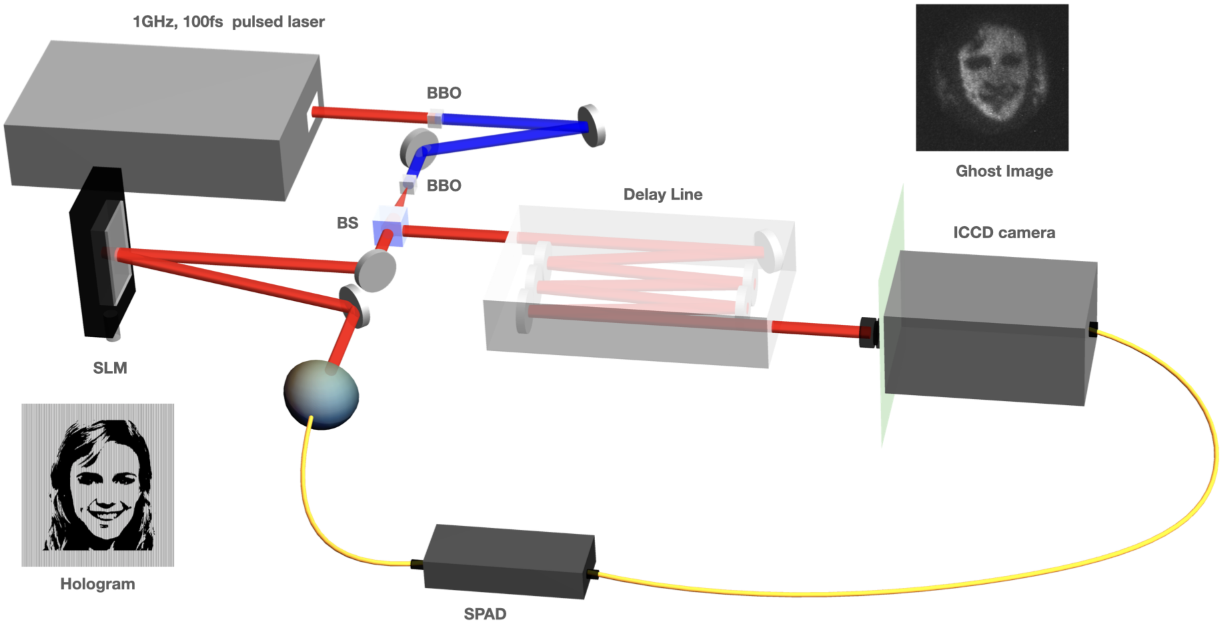

Supplementary Figure S1. Simplified schematic of the experimental setup for Quantum Ghost Imaging. A 1 GHz, 100 fs laser is used to pump a nonlinear crystal (BBO) for second harmonic generation. The second harmonic beam is used to pump a Type-I bismuth triborate (BiBO) crystal for entangled photon pair generation. One of the photons is sent to a Spatial Light Modulator, a liquid crystal device, on which images of human faces are superposed with a diffraction grating. The second photon is sent to a camera through an image preserving delay line where the image of the object is formed. Figure legends: BiBO - 0.5-mm-thick bismuth triborate crystal; BS - Beamsplitter; BS - Beam splitter; SPAD - Single Photon Avalanche Diode; ICCD - Intensified CCD camera.

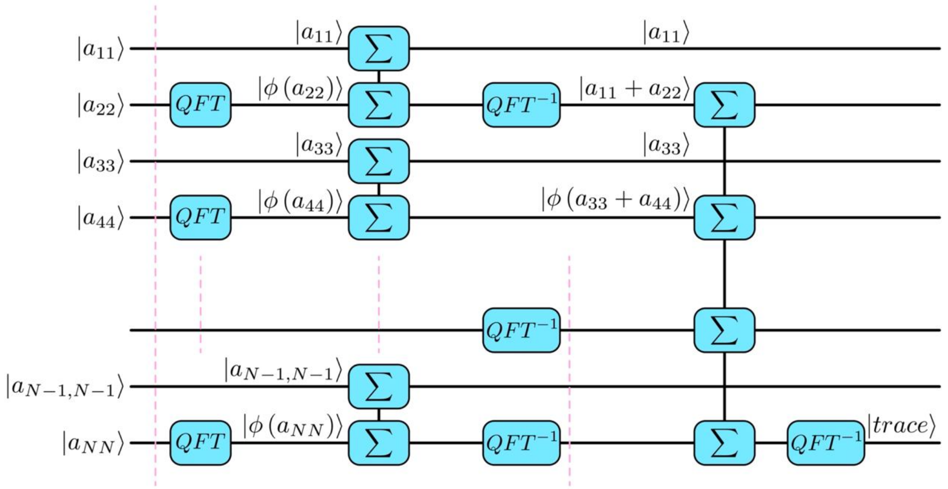

Supplementary Figure S2. Quantum circuit for the trace calculation of sparse matrix.

S2. QUANTUM COMPUTATION OF TRACE

Here, we suggest an adder algorithm [44] to compute the trace of a matrix via adding the diagonal elements of the matrix A. This operator is mainly based on quantum Fourier transform (QFT) and inverse QFT (i.e. QFT^{-1}). The algorithm should process the binary forms of the diagonal. For example, the binary representation of the diagonal elements a_{11} and a_{22} of matrix A are respectively $a_{11} = \alpha_1 2^{n-1} + \alpha_2 2^{n-2} + \ldots + \alpha_n 2^0$ and $a_{22} = \beta_1 2^{n-1} + \beta_2 2^{n-2} + \ldots + \beta_n 2^0$, which are $|a_{11}\rangle = |\alpha_1\rangle \otimes |\alpha_2\rangle \otimes \ldots |\alpha_n\rangle$ and $|a_{22}\rangle = |\beta_1\rangle \otimes |\beta_2\rangle \otimes \ldots |\beta_n\rangle$ in the form of quantum kets. The QFT operation on binary state is QFT$|a\rangle = \frac{1}{\sqrt{N}} \sum_{k=0}^{N-1} e^{\frac{i2\pi ak}{N}} |k\rangle$ and the operation of QFT^{-1} is QFT$^{-1}|k\rangle = \frac{1}{\sqrt{N}} \sum_{a=0}^{N-1} e^{\frac{-i2\pi ak}{N}} |a\rangle$[44]. For simplicity, we introduce a representation for QFT as

$$|\Phi(a)\rangle = \text{QFT}|a\rangle = \frac{1}{\sqrt{N}} \sum_{k=0}^{N-1} e^{\frac{i2\pi ak}{N}} |k\rangle,$$

so, we can write

$$\text{QFT}^{-1}|\Phi(a)\rangle = \text{QFT}^{-1}\text{QFT}|a\rangle = |a\rangle.$$

In order to calculate the trace, we need to add all diagonal elements $|a_{11}\rangle + |a_{22}\rangle + \ldots + |a_{NN}\rangle$ to have the ket include the value of trace as $|a_{11} + a_{22} + \ldots + a_{NN}\rangle$. We introduce the operator Σ that adds two elements a_{11} and a_{22} in the form of $|a_{11} + a_{22}\rangle$ as follows:

$$\Sigma(|a_{11}\rangle|\Phi(a_{22})\rangle) = |\Phi(a_{11} + a_{22})\rangle.$$

Then, after the operation of QFT^{-1} we obtain

$$\text{QFT}^{-1}(|\Phi(a_{11} + a_{22})\rangle) = |a_{11} + a_{22}\rangle.$$

By continuation of this method for the all diagonal elements, the trace can be obtained. The quantum protocol for computation of trace is depicted in Fig. S2, in which the input is $|a_{11}\rangle|a_{22}\rangle \ldots |a_{NN}\rangle$ and the output is $|\Phi(a_{11} + a_{22} + \ldots + a_{NN})\rangle = |\Phi(Tr(A))\rangle$. Finally, by an operation of QFT^{-1} we can get $|Tr(A)\rangle$. The whole process based on QFT and QFT^{-1} has a complexity $\log N$.

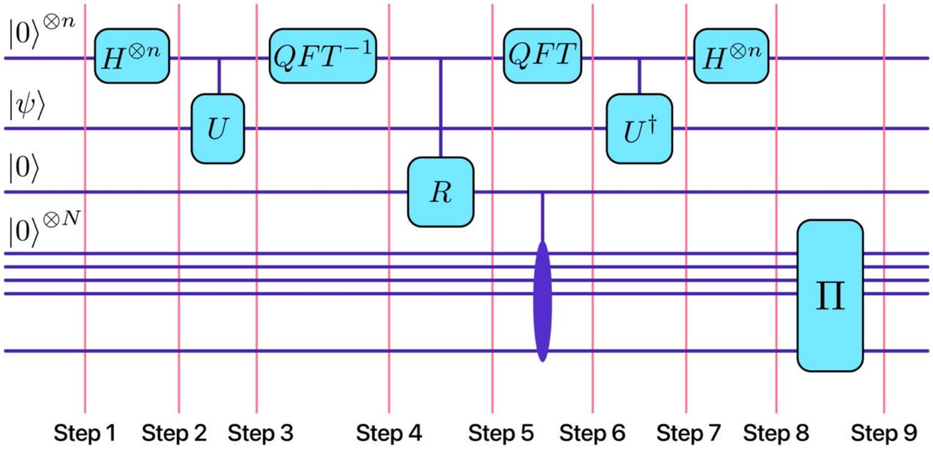

Supplementary Figure S3. Quantum circuit of determinant calculation of sparse matrix

S3. QUANTUM COMPUTATION OF SPARSE MATRIX DETERMINANTS

Our algorithm for computation of determinant is clarified in the following subsections as inputs, algorithm boxes, and algorithm steps:

1. *Inputs*

- Sparse matrix A
- $|0\rangle^{\otimes n}|\Psi\rangle$ as the input in QPE
- $|0\rangle$ as the ancilla for rotation operator
- $|0\rangle^{\otimes N}$ as the memory register for multiplication operator

2. *Algorithm Boxes*

- QPE is the quantum phase estimation subroutine composed of $H^{\otimes n}$, CU (i.e. controlled-U) and inverse quantum Fourier transform (QFT^{-1})
- Rotation operation ($\mathbb{R}$)
- (QPE)$^{-1}$ is the inverse operation of QPE, composed of $H^{\otimes n}$, CU† and quantum Fourier transform (QFT)
- Π is a multiplication operation

The matrix A can be exponentiated as the unitary operator $U = e^{\frac{2\pi i A}{2^n}}$ with logarithmic complexity [7] in which n is the precision. This unitary operator is used in the controlled-U (i.e. CU) part of QPE, and $|\Psi\rangle$ is the superposition of the eigenvectors of A in the form of $|\Psi\rangle = \sum \beta_j |u_j\rangle$. Figure S3 is the representation of the quantum protocol for the computation of matrix determinants. The following steps are based on the steps shown in Fig. S3.

STEP 1:
The initial state of the algorithm is:

$$|0\rangle^{\otimes n}|\Psi\rangle|0\rangle|0\rangle^{\otimes N}. \tag{S1}$$

STEP 2:
After the operation of n Hadamard gates, i.e. $H^{\otimes n}$, we have:

$$\frac{1}{2^{\frac{n}{2}}} \sum_{y_1,\ldots,y_n=0}^{1} |y_1 \ldots y_n\rangle|\Psi\rangle|0\rangle|0\rangle^{\otimes N}. \tag{S2}$$

STEP 3:
In this step, let us apply the controlled-U (CU) operation:

$$\frac{1}{2^{\frac{n}{2}}}\sum_{y=0}^{2^n-1}\sum_{j=1}^{N}\beta_j e^{\frac{2\pi i\lambda_j y}{2^n}}|y\rangle|u_j\rangle|0\rangle|0\rangle^{\otimes N}, \tag{S3}$$

where $y=\sum_{l=1}^{n} y_l 2^{n-l}$ and λ_j's are the eigenvalues of matrix A.

STEP 4:
Here, we apply the inverse Fourier Transform,

$$\frac{1}{2^n}\sum_{y=0}^{2^n-1}\sum_{k=0}^{2^n-1}\sum_{j=1}^{N}\beta_j e^{2\pi i\left(\frac{\lambda_j-k}{2^n}\right)y}|k\rangle|u_j\rangle|0\rangle|0\rangle^{\otimes N}. \tag{S4}$$

For a single k among the all possible values, we have $\lambda_j-k=0$, where $\lambda_j=x=k$. The other terms will be set to zero. Thus, the notation k is changed to the notation x:

$$\frac{1}{2^n}\sum_{y=0}^{2^n-1} e^{2\pi i\left(\frac{\lambda_j-x}{2^n}\right)y}=1 \tag{S5}$$

In this case, the state becomes:

$$\sum_{x=0}^{2^n-1}\sum_{j=1}^{N}\beta_j|\lambda_j\rangle|u_j\rangle|0\rangle|0\rangle^{\otimes N}=\sum_{x_1\dots x_n=0}^{1}\sum_{j=1}^{N}\beta_j|x_1x_2\dots x_n\rangle|u_j\rangle|0\rangle|0\rangle^{\otimes N},$$

where

$$\lambda_j=\sum_{l=0}^{n}x_l 2^{n-l}=2^n\sum_{l=0}^{n}x_l 2^{-l}=2^n\tilde{\lambda}_j\,.$$

STEP 5:
In this step, we apply the rotation operation $\mathbb{R}_l=e^{\frac{i\sigma_y}{2^l}}$, where σ_y is the Pauli matrix y, which acts on the output of QFT^{-1}:

$$\sum_{x_1\dots x_n=0}^{1}\sum_{j=1}^{N}\beta_j|x_1\dots x_n\rangle|u_j\rangle(\sqrt{1-\tilde{\lambda}_j^2}|0\rangle+\tilde{\lambda}_j|1\rangle). \tag{S6}$$

STEP 6:
Applying the quantum Fourier transform (QFT) results in:

$$\frac{1}{2^{\frac{n}{2}}}\sum_{y=0}^{2^n-1}\sum_{j=1}^{N}\beta_j e^{\frac{2\pi i\lambda_j y}{2^n}}|y\rangle|u_j\rangle(\sqrt{1-\tilde{\lambda}_j^2}|0\rangle+\tilde{\lambda}_j|1\rangle). \tag{S7}$$

STEP 7:
In this step, we apply the CU† operator,

$$\frac{1}{2^{\frac{n}{2}}}\sum_{y=0}^{2^n-1}\sum_{j=1}^{N}\beta_j|y\rangle|u_j\rangle(\sqrt{1-\tilde{\lambda}_j^2}|0\rangle+\tilde{\lambda}_j|1\rangle). \tag{S8}$$

STEP 8:
As $|u_j\rangle$'s are known, we repeat the algorithm N times, each time for a specific $|u_j\rangle$, and consequently we obtain the following state:

$$|0\rangle^{\otimes n}|\Psi\rangle\prod_{j=1}^{n}(\sqrt{1-\tilde{\lambda}_j^2}|0\rangle+\tilde{\lambda}_j|1\rangle). \tag{S9}$$

Now, the goal is to measure the multiplication of $\tilde{\lambda}_j$'s in the output of multiplication operation;

$$\prod_{j}^{N}\left(\sqrt{1-\tilde{\lambda}_j^2}|0\rangle+\tilde{\lambda}_j|1\rangle\right)=\left(\sqrt{1-\tilde{\lambda}_1^2}|0\rangle+\tilde{\lambda}_1|1\rangle\right)\ldots\left(\sqrt{1-\tilde{\lambda}_N^2}|0\rangle+\tilde{\lambda}_N|1\rangle\right) \tag{S10}$$

$$=\left(\sqrt{1-\tilde{\lambda}_1^2}\right)\ldots\left(\sqrt{1-\tilde{\lambda}_N^2}\right)|00\ldots0\rangle+\left(\sqrt{1-\tilde{\lambda}_1^2}\right)(\tilde{\lambda}_2)\ldots|0100\ldots0\rangle+\ldots+\overbrace{(\tilde{\lambda}_1)(\tilde{\lambda}_2)\ldots(\tilde{\lambda}_N)}^{\tilde{\lambda}_1\ldots\tilde{\lambda}_N}\overbrace{|111\ldots1\rangle}^{N}. \tag{S11}$$

The coefficient of the state $|\underbrace{11\ldots1}_{N}\rangle$ is the term $\tilde{\lambda}_1\tilde{\lambda}_2\ldots\tilde{\lambda}_N$ in the output of the multiplier, which can be obtained via a weak measurement without collapsing other lines. As $\lambda_j=2^n\tilde{\lambda}_j$, we can obtain the determinant of A (i.e. $\lambda_1\lambda_2\ldots\lambda_N$) via relation $(2^n)^N\tilde{\lambda}_1\tilde{\lambda}_2\ldots\tilde{\lambda}_N=\lambda_1\lambda_2\ldots\lambda_N=det(A)$.

A. Proof of the steps

The proof for each of the eight steps, described above, are given in details in the following expressions.

STEP 2:

$$|\Psi_2\rangle=(H_2^{\otimes n}\otimes\mathbb{I}_N\otimes\mathbb{I}_2\otimes\mathbb{I}_2^{\otimes N})|0\rangle^{\otimes n}|\Psi\rangle|0\rangle|0\rangle^{\otimes N}=\frac{1}{2^{\frac{n}{2}}}\sum_{y=0}^{2^n-1}|y\rangle|\Psi\rangle|0\rangle|0\rangle^{\otimes N}$$

$$=\frac{1}{2^{\frac{n}{2}}}\sum_{y_1\ldots y_n=0}^{1}|y_1...y_n\rangle|\Psi\rangle|0\rangle|0\rangle^{\otimes N} \tag{S12}$$

STEP 3:

$$|\Psi_3\rangle=\prod_{l=1}^{n}(\mathbb{I}_2^{\otimes l-1}\otimes|0\rangle\langle0|\otimes\mathbb{I}_2^{\otimes n-l}\otimes\mathbb{I}_N\otimes\mathbb{I}_2\otimes\mathbb{I}_2^{\otimes N}+\mathbb{I}_2^{\otimes l-1}\otimes|1\rangle\langle1|\otimes\mathbb{I}_2^{\otimes n-l}\otimes U^{2^{n-l}}\otimes\mathbb{I}_2\otimes\mathbb{I}_2^{\otimes N})\frac{1}{2^{\frac{n}{2}}}\times$$

$$\times\sum_{y_1\ldots y_n=0}^{1}|y_1...y_n\rangle\sum_{j=1}^{N}\beta_j|u_j\rangle|0\rangle|0\rangle^{\otimes N}$$

$$=\frac{1}{2^{\frac{n}{2}}}\sum_{y_1\ldots y_n=0}^{1}\sum_{j=1}^{N}\beta_j\prod_{l=1}^{n}(\mathrm{CU})_l|y_1...y_n\rangle|u_j\rangle|0\rangle|0\rangle^{\otimes N}$$

$$=\frac{1}{2^{\frac{n}{2}}}(\sum_{y_1\ldots y_n=0}^{1}\sum_{j=1}^{N}\beta_j\prod_{l=1}^{n}(\delta_{0,y_l}+\delta_{1,y_l}e^{2\pi i\tilde{\lambda}_j2^{n-l}})|y_1...y_n\rangle|u_j\rangle|0\rangle)|0\rangle^{\otimes N}$$

$$=\frac{1}{2^{\frac{n}{2}}}(\sum_{y_1\ldots y_n=0}^{1}\sum_{j=1}^{N}\beta_j\prod_{l=1}^{n}e^{2\pi i\tilde{\lambda}_jy_l2^{n-l}}|y_1...y_n\rangle|u_j\rangle|0\rangle)|0\rangle^{\otimes N}$$

$$=\frac{1}{2^{\frac{n}{2}}}(\sum_{y_1\ldots y_n=0}^{1}\sum_{j=1}^{N}\beta_je^{2\pi i\tilde{\lambda}_j\sum_{l=1}^{n}y_l2^{n-l}}|y_1...y_n\rangle|u_j\rangle|0\rangle)|0\rangle^{\otimes N}$$

$$=\frac{1}{2^{\frac{n}{2}}}\sum_{y=0}^{2^n-1}\sum_{j=1}^{N}\beta_je^{2\pi i\tilde{\lambda}_jy}|y\rangle|u_j\rangle|0\rangle|0\rangle^{\otimes N} \tag{S13}$$

STEP 4:

$$
\begin{aligned}
|\Psi_4\rangle &= (\mathrm{QFT}^{-1}\otimes \mathbb{I}_N \otimes \mathbb{I}_2 \otimes \mathbb{I}_2^{\otimes N})\frac{1}{2^{\frac{n}{2}}}\sum_{y=0}^{2^n-1}\sum_{j=1}^{N}\beta_j e^{2\pi i\tilde{\lambda}_j y}|y\rangle|u_j\rangle|0\rangle|0\rangle^{\otimes N} \\
&= \frac{1}{2^{\frac{n}{2}}}\sum_{y=0}^{2^n-1}\sum_{j=1}^{N}\beta_j e^{2\pi i\tilde{\lambda}_j y}(\mathrm{QFT}^{-1}|y\rangle)|u_j\rangle|0\rangle|0\rangle^{\otimes N} \\
&= \frac{1}{2^{\frac{n}{2}}}\sum_{y=0}^{2^n-1}\sum_{j=1}^{N}\beta_j e^{2\pi i\tilde{\lambda}_j y}(\frac{1}{2^{\frac{n}{2}}}\sum_{k=0}^{2^n-1}e^{-2\pi i\frac{k}{2^n}y}|k\rangle\langle y|y\rangle)|u_j\rangle|0\rangle|0\rangle^{\otimes N} \\
&= \frac{1}{2^n}\sum_{y=0}^{2^n-1}\sum_{k=0}^{2^n-1}\sum_{j=1}^{N}\beta_j e^{2\pi i(\tilde{\lambda}_j-\frac{k}{2^n})y}|k\rangle|u_j\rangle|0\rangle|0\rangle^{\otimes N} \\
&= \frac{1}{2^n}\sum_{y=0}^{2^n-1}\sum_{k=0}^{2^n-1}\sum_{j=1}^{N}\beta_j e^{2\pi i(\frac{\lambda_j-k}{2^n})y}|k\rangle|u_j\rangle|0\rangle|0\rangle^{\otimes N}
\end{aligned} \tag{S14}
$$

$$
|\Psi_4\rangle = \sum_{x=0}^{2^n-1}\sum_{j=1}^{N}\beta_j|\lambda_j\rangle|u_j\rangle|0\rangle|0\rangle^{\otimes N} = \sum_{x_1\ldots x_n=0}^{1}\sum_{j=1}^{N}\beta_j|x_1x_2\ldots x_n\rangle|u_j\rangle|0\rangle|0\rangle^{\otimes N} \tag{S15}
$$

STEP 5:

$$
\begin{aligned}
|\Psi_5\rangle &= \prod_{l=1}^{n}(\mathbb{I}_2^{\otimes n-l+1}\otimes|0\rangle\langle 0|\otimes\mathbb{I}_2^{\otimes l}\otimes\mathbb{I}_N\otimes\mathbb{I}_2\otimes\mathbb{I}_2^{\otimes N}+\mathbb{I}_2^{\otimes n-l+1}\otimes|1\rangle\langle 1|\otimes\mathbb{I}_2^{\otimes l}\otimes\mathbb{I}_N\otimes\mathbb{R}_{n-l})\otimes\mathbb{I}_2^{\otimes N}\times \\
&\times\sum_{x_1\ldots x_n=0}^{1}|x_1\ldots x_n\rangle\sum_{j=1}^{N}\beta_j|u_j\rangle|0\rangle \\
&= \sum_{x_1\ldots x_n=0}^{1}\sum_{j=1}^{N}\beta_j\prod_{l=1}^{n}(\mathrm{CR})_{n-l+1}|x_1\ldots x_n\rangle|u_j\rangle|0\rangle|0\rangle^{\otimes N} \\
&= \sum_{x_1\ldots x_n=0}^{1}\sum_{j=1}^{N}\beta_j|x_1\ldots x_n\rangle|u_j\rangle(\prod_{l=1}^{n}(\delta_{0,x_{n-l+1}}+\delta_{1,x_{n-l+1}}e^{\frac{i\sigma_y}{2^{n-l+1}}})|0\rangle)|0\rangle^{\otimes N} \\
&= \sum_{x_1\ldots x_n=0}^{1}\sum_{j=1}^{N}\beta_j|x_1\ldots x_n\rangle|u_j\rangle(\prod_{l=1}^{n}e^{i\sigma_y x_{n-l+1}2^{-(n-l+1)}}|0\rangle)|0\rangle^{\otimes N} \\
&= \sum_{x_1\ldots x_n=0}^{1}\sum_{j=1}^{N}\beta_j|x_1\ldots x_n\rangle|u_j\rangle(e^{i\sigma_y\sum_{l=1}^{n}x_{n-l+1}2^{-(n-l+1)}}|0\rangle)|0\rangle^{\otimes N} \\
&= \sum_{x_1\ldots x_n=0}^{1}\sum_{j=1}^{N}\beta_j|x_1\ldots x_n\rangle|u_j\rangle(e^{i\sigma_y\tilde{\lambda}_j}|0\rangle)|0\rangle^{\otimes N}.
\end{aligned} \tag{S16}
$$

STEP 6:

$$
\begin{aligned}
|\Psi_6\rangle &= (\mathrm{QFT}\otimes \mathbb{I}_N \otimes \mathbb{I}_2 \otimes \mathbb{I}_2^{\otimes N}) \sum_{x_1...x_n=0}^{1}\sum_{j=1}^{N}\beta_j |x_1...x_n\rangle|u_j\rangle(\sqrt{1-\tilde{\lambda}_j^2}|0\rangle+\tilde{\lambda}_j|1\rangle)\\
&= (\mathrm{QFT}\sum_{x_1...x_n=0}^{1}|x_1...x_n\rangle)\sum_{j=1}^{N}\beta_j|u_j\rangle(\sqrt{1-\tilde{\lambda}_j^2}|0\rangle+\tilde{\lambda}_j|1\rangle)\\
&= (\frac{1}{2^{\frac{n}{2}}}\sum_{y=0}^{2^n-1}e^{2\pi i\frac{k}{2^n}y}|y\rangle\langle k|\sum_{x_1...x_n=0}^{1}|x_1...x_n\rangle)\sum_{j=1}^{N}\beta_j|u_j\rangle(\sqrt{1-\tilde{\lambda}_j^2}|0\rangle+\tilde{\lambda}_j|1\rangle)\\
&= (\frac{1}{2^{\frac{n}{2}}}\sum_{y=0}^{2^n-1}e^{2\pi i\frac{k}{2^n}y}|y\rangle\sum_{x_1...x_n=0}^{1}\langle k|x_1...x_n\rangle)\sum_{j=1}^{N}\beta_j|u_j\rangle(\sqrt{1-\tilde{\lambda}_j^2}|0\rangle+\tilde{\lambda}_j|1\rangle)\\
&= (\frac{1}{2^{\frac{n}{2}}}\sum_{y=0}^{2^n-1}e^{2\pi i\frac{k}{2^n}y}|y\rangle\sum_{x_1...x_n=0}^{1}\delta_{k,x_1...x_n})\sum_{j=1}^{N}\beta_j|u_j\rangle(\sqrt{1-\tilde{\lambda}_j^2}|0\rangle+\tilde{\lambda}_j|1\rangle)\\
&= (\frac{1}{2^{\frac{n}{2}}}\sum_{y=0}^{2^n-1}e^{2\pi i\frac{k}{2^n}y}|y\rangle\delta_{k,\lambda_j})\sum_{j=1}^{N}\beta_j|u_j\rangle(\sqrt{1-\tilde{\lambda}_j^2}|0\rangle+\tilde{\lambda}_j|1\rangle)\\
&= \frac{1}{2^{\frac{n}{2}}}\sum_{y=0}^{2^n-1}e^{2\pi i\frac{\lambda_j}{2^n}y}|y\rangle\sum_{j=1}^{N}\beta_j|u_j\rangle(\sqrt{1-\tilde{\lambda}_j^2}|0\rangle+\tilde{\lambda}_j|1\rangle)\\
&= \frac{1}{2^{\frac{n}{2}}}\sum_{y=0}^{2^n-1}\sum_{j=1}^{N}\beta_j e^{2\pi i\frac{\lambda_j}{2^n}y}|y\rangle|u_j\rangle(\sqrt{1-\tilde{\lambda}_j^2}|0\rangle+\tilde{\lambda}_j|1\rangle)
\end{aligned}
\tag{S17}
$$

STEP 7:

$$
\begin{aligned}
|\Psi_7\rangle &= \prod_{l=1}^{n}(\mathbb{I}_2^{\otimes l-1}\otimes|0\rangle\langle 0|\otimes\mathbb{I}_2^{\otimes n-l}\otimes\mathbb{I}_N\otimes\mathbb{I}_2\otimes\mathbb{I}_2^{\otimes N}+\mathbb{I}_2^{\otimes l-1}\otimes|1\rangle\langle 1|\otimes\mathbb{I}_2^{\otimes n-l}\otimes(U^\dagger)^{2^{n-l}}\otimes\mathbb{I}_2\otimes\mathbb{I}_2^{\otimes N})\times\\
&\times\frac{1}{2^{\frac{n}{2}}}\sum_{y=0}^{2^n-1}\sum_{j=1}^{N}\beta_j e^{2\pi i\frac{\lambda_j}{2^n}y}|y\rangle|u_j\rangle(\sqrt{1-\tilde{\lambda}_j^2}|0\rangle+\tilde{\lambda}_j|1\rangle)\\
&= \frac{1}{2^{\frac{n}{2}}}\sum_{y_1...y_n=0}^{1}\sum_{j=1}^{N}\beta_j e^{2\pi i\frac{\lambda_j}{2^n}y}\prod_{l=1}^{n}(\mathrm{CU}^\dagger)_l|y_1...y_n\rangle|u_j\rangle(\sqrt{1-\tilde{\lambda}_j^2}|0\rangle+\tilde{\lambda}_j|1\rangle)\\
&= \frac{1}{2^{\frac{n}{2}}}(\sum_{y_1...y_n=0}^{1}\sum_{j=1}^{N}\beta_j e^{2\pi i\frac{\lambda_j}{2^n}y}\prod_{l=1}^{n}(\delta_{0,y_l}+\delta_{1,y_l}e^{-2\pi i\lambda_j 2^{n-l}})|y_1...y_n\rangle|u_j\rangle(\sqrt{1-\tilde{\lambda}_j^2}|0\rangle+\tilde{\lambda}_j|1\rangle)\\
&= \frac{1}{2^{\frac{n}{2}}}(\sum_{y_1...y_n=0}^{1}\sum_{j=1}^{N}\beta_j e^{2\pi i\frac{\lambda_j}{2^n}y}\prod_{l=1}^{n}e^{-2\pi i\lambda_j y_l 2^{n-l}}|y_1...y_n\rangle|u_j\rangle(\sqrt{1-\tilde{\lambda}_j^2}|0\rangle+\tilde{\lambda}_j|1\rangle)\\
&= \frac{1}{2^{\frac{n}{2}}}(\sum_{y_1...y_n=0}^{1}\sum_{j=1}^{N}\beta_j e^{2\pi i\frac{\lambda_j}{2^n}y}e^{-2\pi i\lambda_j\sum_{l=1}^{n}y_l 2^{n-l}}|y_1...y_n\rangle|u_j\rangle(\sqrt{1-\tilde{\lambda}_j^2}|0\rangle+\tilde{\lambda}_j|1\rangle)\\
&= \frac{1}{2^{\frac{n}{2}}}(\sum_{y_1...y_n=0}^{1}\sum_{j=1}^{N}\beta_j e^{2\pi i\frac{\lambda_j}{2^n}y}e^{-2\pi i\tilde{\lambda}_j y}|y_1...y_n\rangle|u_j\rangle(\sqrt{1-\tilde{\lambda}_j^2}|0\rangle+\tilde{\lambda}_j|1\rangle)\\
&= \frac{1}{2^{\frac{n}{2}}}\sum_{y=0}^{2^n-1}\sum_{j=1}^{N}\beta_j e^{\frac{2\pi i}{2^n}(\lambda_j-\lambda_j)y}|y\rangle|u_j\rangle(\sqrt{1-\tilde{\lambda}_j^2}|0\rangle+\tilde{\lambda}_j|1\rangle)\\
&= \frac{1}{2^{\frac{n}{2}}}\sum_{y=0}^{2^n-1}\sum_{j=1}^{N}\beta_j|y\rangle|u_j\rangle(\sqrt{1-\tilde{\lambda}_j^2}|0\rangle+\tilde{\lambda}_j|1\rangle).
\end{aligned}
\tag{S18}
$$

STEP 8:

$$|\Psi_8\rangle = (H_2^{\otimes n} \otimes \mathbb{I}_N \otimes \mathbb{I}_2 \otimes \mathbb{I}_2^{\otimes N}) \frac{1}{2^{\frac{n}{2}}} \sum_{y=0}^{2^n-1} \sum_{j=1}^{N} \beta_j |y\rangle |u_j\rangle (\sqrt{1-\tilde{\lambda}_j^2}|0\rangle + \tilde{\lambda}_j|1\rangle)$$
$$= |0\rangle^{\otimes n} \sum_{j=1}^{N} \beta_j |u_j\rangle (\sqrt{1-\tilde{\lambda}_j^2}|0\rangle + \tilde{\lambda}_j|1\rangle) \tag{S19}$$

$$\prod_{j}^{N} \left(\sqrt{1-\tilde{\lambda}_j^2}|0\rangle + \tilde{\lambda}_j|1\rangle\right) = \left(\sqrt{1-\tilde{\lambda}_1^2}|0\rangle + \tilde{\lambda}_1|1\rangle\right) \dots \left(\sqrt{1-\tilde{\lambda}_N^2}|0\rangle + \tilde{\lambda}_N|1\rangle\right)$$
$$= \left(\sqrt{1-\tilde{\lambda}_1^2}\right) \dots \left(\sqrt{1-\tilde{\lambda}_N^2}\right) |00\dots0\rangle + \left(\sqrt{1-\tilde{\lambda}_1^2}\right) (\tilde{\lambda}_2) \dots |0100\dots0\rangle + \dots + \overbrace{(\tilde{\lambda}_1)(\tilde{\lambda}_2)\dots(\tilde{\lambda}_N)}^{\tilde{\lambda}_1\dots\tilde{\lambda}_N} \overbrace{|111\dots1\rangle}^{N} \tag{S20}$$
